# Psychotherapeutic approaches to non-suicidal self-injury in adolescents

**DOI:** 10.1186/1753-2000-6-14

**Published:** 2012-03-30

**Authors:** Jason J Washburn, Sarah L Richardt, Denise M Styer, Michelle Gebhardt, K R Juzwin, Adrienne Yourek, Delia Aldridge

**Affiliations:** 1 Alexian Brothers Behavioral Health Hospital, Center for Evidence-Based Practice, 1650 Moon Lake Blvd, Hoffman Estates, IL 60169, USA; 2 Department of Psychiatry and Behavioral Sciences, Northwestern University Feinberg School of Medicine, 710 N. Lake Shore Drive, Chicago, IL 60611, USA; 3 College of Psychology and Behavioral Sciences, Argosy University, 999 N. Plaza Drive, Schaumburg, IL, 60173, USA

**Keywords:** Non-suicidal self-injury, Psychotherapy, Treatment, Adolescent, Review

## Abstract

Non-suicidal self-injury (NSSI) among adolescents is gaining increasing attention in both clinical and scientific arenas. The lifetime prevalence of NSSI is estimated to vary between 7.5% to 8% for preadolescents, increasing to between 12% and 23% for adolescents. Despite the prevalence and the increasing interest in NSSI, few psychotherapeutic treatments have been designed specifically for NSSI, and no treatments have been evaluated specifically for the treatment of NSSI among adolescents. Consequently, child and adolescent clinicians are left with little evidence-based guidance for treating this challenging population. To provide some guidance, evaluations of treatments for adults with NSSI and for adolescents with related conditions, such as deliberate self-harm and borderline personality disorder, are reviewed. Clinical guidelines and resources are also discussed to assist with the gaps in the knowledge base for treatment of NSSI among adolescents.

## Introduction

**Figure 1:**
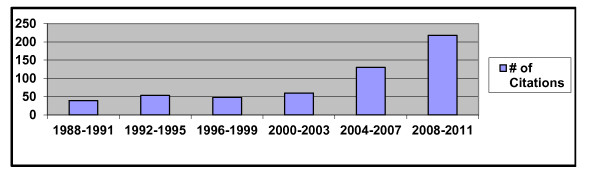
Number (#) of Citations for “Self-Injury” for Children and Adolescents (1988–2011).

Clinical and scientific interest in self-injury among children and adolescents has increased dramatically in the last decade. Figure 1 provides results of a simple citation search using the search term “self-injury” in PsychINFO®, and limiting the results to children and adolescents. The number of citations involving “self-injury” has increased steadily in the last decade, with citations increasing by five times from 1988–1991 to 2008–2011. An examination of the specific citations over this time period indicates that until recently, the majority of citations focused on self-injury involving either suicidal self-injury or stereotypic self-injurious behavior among children and adolescents with intellectual or developmental disabilities. More recent citations, however, focus increasingly on *non-suicidal self-injury* among children and adolescents without intellectual or developmental delays. In contrast to suicidal self-injury or stereotypic self-injurious behavior, non-suicidal self-injury (NSSI) is the deliberate, self-inflicted damage of body tissue that induces bleeding, bruising, or pain, but is absent of evidence for suicidal intent and is not for purposes that are social sanctioned (e.g., tattooing, piercing) [1].

The lifetime prevalence of NSSI is estimated to vary between 7.5% to 8% for preadolescents [2,3], and increases to 12% to 23% for adolescents [4,5]. Among clinical populations of adolescents, the prevalence rate of NSSI varies even more dramatically, with rates between 12% and 82% reported in the literature [6,7]. A recent study of adolescents with treatment resistant depression found that NSSI was more common than suicide attempts (38% vs. 23%), underscoring the prevalence of this disorder among adolescents seen in clinical settings [8]. Considering that the typical age of onset for NSSI is between 11 and 15 years of age for adolescents who engage in NSSI [2,9,10], most research on NSSI in youth – including this review – focuses on adolescents rather than children.

### Evidence-based review of psychotherapeutic treatments for NSSI

The literature search described above was repeated using the terms “self-injury” or “self-harm” combined with the terms “treatment” or “therapy” in PsychINFO®, PubMed, and ClinicalTrials.gov databases. Results of this refined search indicate that despite an increased interest in NSSI in the literature, few psychotherapeutic treatments have been designed and evaluated specifically for NSSI [11]. Of grave concern is that no treatments have been designed and evaluated specifically for NSSI among adolescents. The dearth of interventions for NSSI among adolescents may be due to the relatively recent interest and recognition of the problem of NSSI among this age group [12], and may improve with the adoption of NSSI as a psychiatric disorder in the fifth edition of the Diagnostic and Statistical Manual of Mental Disorder [13].

The lack of empirically supported treatments for NSSI, however, presents a dire situation for the clinician who is left to treat youth with NSSI without reference to evidence-based strategies. Guidance on how to treat adolescents presenting with NSSI may be obtained from studies of adults with NSSI, as well as studies of adolescents and adults with related conditions or disorders. For example, a handful of studies of have evaluated psychosocial interventions for *deliberate self-harm* (DSH). DSH typically refers to self-injury that can be suicidal and/or non-suicidal [1].

Cognitive and behavioral therapies (CBT) show the most promise in treating NSSI across various settings [14]. A form of CBT, Problem-Solving Therapy [15], was one of the first treatments for DSH to be evaluated using randomized controlled trials. Problem-Solving Therapy involves training in the skills and attitudes necessary to promote active problem solving [16]. Treatment with Problem-Solving Therapy focuses on accomplishing the following goals: (1) Developing or enhancing a positive problem orientation and decreasing a negative orientation; (2) Training in rational problem solving (i.e., defining and formulating the problem, generating alternative solutions, making a decision, and solution verification); and (3) reducing avoidance of problem solving, as well impulsive and careless decision making [17]. Within this model, NSSI is conceptualized as a dysfunctional solution to problems, with improved problem solving attitudes and skills leading to decreased reliance on NSSI to cope.

Evaluations of Problem-Solving Therapy with patients with DSH suggest promise as a treatment, but with limitations. An early meta-analysis found a trend toward reduction of DSH with therapies focused on problem solving, but when compared to control conditions, the difference was not statistically significant [18]. A later meta-analysis of six randomized controlled studies, four of which included at least some older adolescents (15–17 years old), found Problem-Solving Therapy to be superior to control conditions in reducing depression and hopelessness, and in improving problem solving [19]. Unfortunately, this meta-analysis did not directly examine the effects of these treatments on reduction of actual DSH. A recent study of group-based Problem-Solving Therapy for adult females who engaged in self-poisoning also found preliminary evidence for improvement with depression, hopelessness, suicidal ideation, and social problem solving, but also failed to show a significant difference between the control and treatment group; indeed, neither group evidenced DSH during the two-month follow-up [20].

The lack of consistent results of Problem-Solving Treatments for DSH has led some to argue that treatment must integrate strategies beyond problem solving skills and attitudes to be effective in treating DSH [14]. Manual-Assisted Cognitive-behavioral Therapy (MACT) for DSH was developed as just such a treatment. MACT is a brief therapy for DSH that integrates problem-solving therapy with cognitive techniques and relapse prevention strategies. An early pilot study of MACT, which included some adolescents, found a lower rate of DSH for MACT when compared to treatment as usual [21]. Consistent with several of the other problem-solving therapies, however, the difference was not statistically significant. A multi-site randomized controlled study of MACT was subsequently conducted with 480 people, including some adolescents (ages 16 and 17). Although the results supported the cost-effectiveness of MACT over treatment as usual, no significant effect was found on repeated DSH [22]. Further analyses indicated that for participants with borderline personality disorder, MACT was associated with *increased* costs when compared to treatment as usual [23]. A newly developed version of MACT, the “Cutting Down” program, has recently been developed and piloted specifically for adolescents with DSH [24]. Although findings from this single-group open trial study of 24 adolescents suggest promise in reducing DSH, these results need to be tested under more rigorous conditions (e.g., control or treatment comparison group, randomized assignment, larger sample), especially given the disappointing findings of prior MACT treatments when evaluated in randomized controlled trials [22,23].

A group therapy for DSH, Developmental Group Psychotherapy, has also been extensively evaluated with adolescents. This therapeutic approach combines problem-solving skills training with aspects of Dialectical Behavior Therapy (described below) and psychodynamic therapy. An initial evaluation of this treatment found a reduction in repeated DSH when compared to treatment as usual [25]. A replication of this treatment, however, failed to find improvement in DSH over treatment as usual [26]. An additional large-scale (n = 366 adolescents) replication of the Developmental Group Psychotherapy treatment also failed to show the superiority of the treatment over treatment as usual, nor was it cost-effective over treatment as usual [27].

The Treatment of SSRI-Resistant Adolescent Depression (TORDIA) study is the only study that we found that evaluated NSSI separately from suicidal self-injury as a treatment outcome [28]. The TORDIA study included adolescents, ages 12–18, who had a diagnosis of major depressive disorder but did not respond to a selective-serotonin uptake inhibitor (SSRI). Treatment arms included antidepressant medication (venlafaxine or a different SSRI), with or without CBT. The CBT arm included cognitive restructuring and behavior activation components, skills training in emotion regulation, social skills, and problem-solving, as well as parent–child sessions to improve support, decrease criticism, and improve family communication and problem-solving. Approximately one-third of the sample also had a history of NSSI [28]. As such, this represents a unique study in that it is the only treatment study for adolescents that did not collapse NSSI and suicidal self-injury into DSH.

Overall results of the TORDIA study at 12 weeks of treatment indicated that a combination of CBT with medication (either venlafaxine or a different SSRI) was more efficacious in reducing depressive symptoms than just switching to a different medication [28]. Problem-solving and social skills appeared to be the most effective components of the CBT intervention in this study [29]. The superiority of CBT and medication over medication alone, however, was not sustained at the 24-week follow-up [30]. Further, there were no differences in the rate of NSSI events across the various treatments, including medication and CBT [31]. The findings of this study suggest that treatments that may be effective for a condition related to NSSI may not adequately address NSSI [31].

More promising findings are found in a study examining the efficacy of a 12-session CBT intervention for DSH [32]. This study included 82 individuals who engaged in DSH, including adolescents (age 15–17) and adults, randomized to either an adjunctive CBT intervention or to treatment as usual. In contrast to the TORDIA study, this adjunctive CBT intervention was developed to specifically identify and modify the mechanisms that maintain DSH. Specifically, this CBT treatment directly assessed the most recent episode of DSH, examined emotional, cognitive, and behavioral contributions to the maintenance of DSH, and addressed these maintaining factors using cognitive and behavioral strategies. This focused, adjunctive CBT intervention for DSH was found to be superior to treatment as usual in reducing episodes of DSH at the 9-month follow-up. The authors suggest that CBT was effective in reducing DSH because it actively targeted the depressive symptoms, suicidal cognitions, and problem-solving deficits that maintained DSH.

The form of CBT with that has the most evidence supporting a reduction in DSH is Dialectical Behavior Therapy (DBT). DBT combines skills-training, exposure and response prevention, contingency management, problem-solving training, and cognitive modification strategies with mindfulness, validation, and acceptance practices [33]. It is important to note, however, that DBT was not designed to treat DSH, but instead was designed to treat borderline personality disorder, which often includes DSH. Randomized and non-randomized studies indicate that DBT is effective in adult patients with borderline personality disorder for a range of outcome variables, including DSH [34-36].

DBT has been adapted for use with adolescents with numerous problem behaviors, including NSSI and suicidal self-injury [37-40]. Studies have also examined the adaptation of DBT for incarcerated male [41] and female [42] adolescents, as well as for children [43]. Despite over a decade of articles on DBT for adolescents, there have been no randomized control studies of DBT in adolescents [44]. Indeed, a 2009 review [45] found only three non-randomized studies of DBT with adolescents that included a comparison group [42,46,47]. Available evidence from quasi-experimental and pre-post designs suggests that DBT for adolescents may be helpful in reducing hospitalization, suicidal ideation, and treatment dropout; however, support for reducing NSSI is limited [38,45]. For example, a feasibility study of DBT on an inpatient unit found that DSH decreased for the DBT group as well as for the treatment as usual group at follow-up [46]. In summary, DBT is an effective form of treatment for NSSI and suicidal self-injury among adults with borderline personality disorder, and therefore holds great promise for treatment of NSSI among adolescents [48]. Empirical support for the application of DBT to adolescents with NSSI, however, remains limited.

Other variations of CBT and non-CBT treatments for DSH have also been explored in the literature. For instance, multisystemic therapy has been evaluated as an alternative to hospitalization for youth engaging in DSH. Multisystemic therapy was originally developed as a treatment for antisocial youth [49] and has been adapted for use with youth in emotional and behavioral crises [50]. Multisystemic therapy is a family-based treatment that is grounded in a social-ecological model, focusing interventions on the multiple systems that maintain youths’ problem behavior [49]. In a randomized trial of youth presenting in psychiatric crisis, multisystemic therapy demonstrated superiority to hospitalization in decreasing DSH, as rated by parents on the Child Behavior Checklist [51]. Because the MST group had higher DSH at baseline than the hospitalization group, however, it wasn’t possible to rule out regression to the mean as an explanation for the treatment effect. Further, no treatment effect of MST was found for depressive affect, hopelessness, and suicidal ideation.

Other variations of treatments for NSSI and DSH have been evaluated with adults, but not adolescents. Emotional regulation group therapy [52], a 14-week adjunctive therapy for NSSI uses strategies from DBT and Acceptance and Commitment therapy. This group treatment has been shown to reduce NSSI in adult women with subthreshold or threshold BPD [53,54], although more studies are needed to confirm the findings. Psychodynamic approaches, including interpersonal psychodynamic psychotherapy [55], mentalization-based therapy [56], object-relations psychodynamic psychotherapy [57], and transference-focused psychotherapy [58] have also been studied for adults with DSH. Interpersonal Therapy for Depressed Adolescents, an efficacious treatment for depressed adolescents [59], has been adapted for use with adolescents with NSSI (ClinicalTrials.gov Identifier: NCT00401102), although results from the randomized controlled trial have yet to be published.

Another treatment currently under evaluation is the Treatment for Non-Suicidal Self-Injury in Young Adults (T-SIB; ClinicalTrials.gov Identifier: NCT01018433). The 9-session T-SIB intervention was designed specifically to treat NSSI among young adults, ages 18 to 29 years, and includes motivational enhancement pre-treatment strategies, functional analysis, and skill training for problem-solving, distress tolerance, cognitive distortions, and interpersonal skills. Although this study is ongoing and no findings have been published, preliminary results support the feasibility, acceptability, and efficacy of the time-limited T-SIB intervention for young adults who engage in NSSI [60].

Finally, preliminary evidence suggests that exercise may be a promising treatment for addressing the urges to engage in NSSI behavior. Exercise or participation in sports has been reported as one of the most helpful strategies to resist urges to engage in NSSI [61]. A single-case, quasi-experimental study of a young adult with a 13-year history of NSSI demonstrated that urge and frequency of NSSI significantly declined with the use of physical exercise [62]. Further research is needed to understand the efficacy of exercise and physical activity among adolescents with NSSI.

In summary, little research has examined the efficacy of treatments designed specifically for adolescents with NSSI. Most of the available studies have focused on DSH instead of NSSI, making it difficult to understand what exactly the treatment is addressing. Further, many of the studies have examined adolescents along with adults; only a handful of studies have focused specifically on adolescents. While variations of CBT enjoy the greatest support in the literature, that support is not consistent when focusing on adolescents with NSSI.

### Clinical guidelines for psychotherapuetic approaches to NSSI

The prior review highlights the dearth of psychotherapeutic treatments designed specifically for adolescents with NSSI. Even without the guidance of empirically-supported treatments for NSSI, clinicians must still treat adolescents with NSSI. Consistent with an evidence-based practice model [63], clinicians can consult clinical guidelines or practice standards in the absence of empirically-supported treatments.

Some national guidelines have been developed for DSH; again, NSSI and suicidal self-injury have been combined in most of these guidelines. An exception is the Mental Health First Aid Training and Research Program out of the University of Melbourne, which provides clinical guidelines for how to respond to someone who has engaged in NSSI, including brief scripts on how to talk to someone engaging in NSSI, what to do if witnessing someone engaging in NSSI, obtaining professional help, and keeping someone safe who is engaging in NSSI [64].

In 2004, the National Institute for Health and Clinical Excellence (NICE; http://www.nice.org.uk) in the United Kingdom published a clinical guideline for DSH. Consistent with the DSH literature, the NICE guidelines are not specific to NSSI, defining self-harm as “self-poisoning or injury, irrespective of the apparent purpose of the act” (p.7). Further only a small section of the NICE guidelines focus on psychotherapeutic interventions, and little guidance is provided to the type of interventions that should be provided. Indeed, the NICE guidelines only reference the need for at least 3 months of “an intensive therapeutic intervention” for people at risk for repetitive self-harm. DBT is recommended for consideration, but only for people with self-harm and a diagnosis of borderline personality disorder.

In 2009, the Royal Australian & New Zealand College of Psychiatrists (RANZCP) published clinical practice guidelines for “self-harm” (http://www.ranzcp.org/resources/clinical-practice-guidelines.html), conflating suicidal and non-suicidal self-injury. The RANZCP guidelines provide some additional details with regard to recommended treatment approaches for self-harm than the NICE guidelines. For example, they list the following treatment goals for self-harm: Treat associated mental illness; Prevent future self-harm; Improve coping skills; Reduce distress; Prevent suicide; Extend the time between self-harm; Reduce injury severity; and Help your family to help you. The guidelines also list the therapeutic approaches that have been shown to be efficacious for DSH and depression more broadly, such as CBT, DBT, Problem-Solving Therapy, and Interpersonal Therapy.

In the past five years, several summary articles and books have been published by established researchers and clinical experts in the area of NSSI that provide more detailed guidelines for the clinician treating adolescents with NSSI [7,14,65-67]. In light of the paucity of empirically supported treatments for NSSI, these recent publications provide guidance for clinicians treating NSSI by integrating the available evidence with clinical consensus. Although a comprehensive review and integration of these recommendations is beyond the scope of this article, some examples of common recommendations are listed below:

· Assessment of NSSI is critical for understanding and treating NSSI. At a minimum, assessment of NSSI should include an understanding of current and past NSSI behavior (types, methods, locations, frequency, age of onset, severity, urges to self-injure), delineation of biopsychosocial risk and protective factors for NSSI, a comprehensive suicide risk assessment, assessment of co-occurring disorders (especially depression, substance abuse, eating disorders, impulse control disorders, posttraumatic stress disorder), and examination of the context and functions of NSSI [65-68]. Several measures are available to assist in an assessment of NSSI, such as the Self-Injurious Thoughts and Behaviors Interview [69], the Ottawa Self-Injury Inventory [70], the Suicide Attempt Self-Injury Interview [71], the Deliberate Self-Harm Inventory [72], the Inventory of Statements about Self-Injury [73,74], the Functional Assessment of Self-Mutilation [75], and the Alexian Brothers Urge to Self-Injure Scale [76].

· Motivational enhancement strategies may be necessary for effective treatment, both prior to and throughout treatment. Although motivational approaches have been proposed for NSSI [68,77], motivational interventions have not been specifically evaluated for adolescents with NSSI.

· Cognitive and behavioral interventions offer the most promise in providing therapy to adolescents with NSSI [65-68]. For example, cognitive strategies, such as Socratic questioning and thought records, address self-derogatory and distorted beliefs about NSSI. Behavioral strategies, such as contingency management and behavioral activation, address environmental factors that maintain NSSI. Dialectical strategies, such as acceptance and tolerance of distress, may address urges to engage in NSSI. Interpersonal approaches may also be helpful in understanding and modifying maladaptive interpersonal styles [68].

· Skills training is likely to be central to the treatment of NSSI. Training should focus on improving emotion regulation, problem-solving, interpersonal, and communication skills [65-68].

· Treatment may need to focus on physical factors. Body image concerns as well as alienation from the body may need to be addressed directly for some individuals with NSSI. Further, physical self-care and exercise hold promise as important components to treatment [66,68].

· Understanding and addressing social contagion with NSSI may be prudent, especially when providing group-based treatment or working with an adolescent’s school [65].

· So-called “contracts for safety” or “no-harm agreements” are either ineffective or harmful, and treatment should instead focus on using contingency management strategies and relapse prevention plans [65,66].

## Conclusions

A 2008 review of the literature on DSH commissioned by the Scottish Government concludes that “[p]opulations which are particularly poorly served by the available literature are people engaged in (currently) non-fatal self-harm, in particular self-cutting; people at either end of the age spectrum (those younger than 15 or older than 65); and people from social, cultural and ethnic minority populations” (p. 3) [78]. This brief review supports this statement; the evidence base for the treatment of adolescents with NSSI is plagued by large gaps in our knowledge. Indeed, to date, no treatments have been specifically designed and evaluated for adolescents engaging in clinically-significant levels of NSSI.

Existing treatments that may be relevant to NSSI tend to focus on adults instead of adolescents, or on depression or borderline personality disorder instead of NSSI. Further, most treatments to date have confused the results of their evaluations by combining NSSI and suicidal self-injury into DSH. The lack of interventions specifically for NSSI is likely due to the conflation of NSSI with other constructs, such as considering NSSI the exclusive domain of borderline personality disorder, or attempting to treat both NSSI and suicidal self-injury as DSH. This last point is particularly concerning and must be remediated in future research. The best available evidence suggests that combining NSSI and suicidal self-injury into more broad and vague constructs like DSH obfuscates two distinct albeit related constructs [13,79,80]. Although concerns about the difficulty of assessing the intent of self-injury still appear to influence decisions to study DSH instead of NSSI in treatment studies [81], several research and clinical instruments are now available to effectively assess for NSSI separate from suicidal self-injury [82-84]. Given the possibility that NSSI will be identified as a distinct disorder in the DSM-V [80], it is critical that the literature begin to focus on NSSI separate from suicidal self-injury [13].

In their review of the literature on adolescent suicide, Miller, Rathus, and Linehan (2007) argue that there is a lack of support for treating suicidal behavior by treating disorders associated with suicidal behavior, such as depression. Although the data are limited, we expect this to be true for NSSI as well. NSSI is likely to require specific psychotherapeutic interventions, beyond the treatment of depression and/or suicidality [13,31,85]. Further, while treatments for borderline personality disorder are likely to be helpful in reducing NSSI in adolescents with these personality characteristics, it is unknown if intensive treatments for borderline personality disorder, such as DBT, are equally effective or even necessary for adolescents with NSSI who don’t have a personality disorder.

In addition to developing treatments for adolescents with NSSI, we must develop dissemination pipelines to move evidence-based treatments out to practicing clinicians. Training clinicians in how to treat adolescents with NSSI is likely to be as great of an obstacle as creating the treatments in the first place. A recent study evaluating the effectiveness of DBT for adults with borderline personality disorder using routine community treatment settings found that therapists who received more intensive training had better outcomes than therapists who only received basic training [86]. Of note, the inferior “basic” training involved *four full days*, a time commitment that, although inferior to the more intensive training discussed in the study, may be unrealistic for many practicing clinicians.

Finally, most of the psychotherapeutic approaches to NSSI discussed in the literature focus on outpatient psychotherapy, with little focus on acute forms of treatment, such as inpatient, partial hospitalization, or residential treatment. Given the strong associations between NSSI, suicidal self-injury and suicide, developing effective psychotherapeutic interventions at acute levels of care is critical. Two recent studies found that NSSI was a stronger predictor of future suicide attempts than prior suicide attempts among adolescents with depression [8,87]. It is therefore likely that a substantial proportion of adolescents presenting to an acute level of care for suicidal behavior will also have either historical or current risk for NSSI. Effective approaches for the management and treatment of NSSI in acute levels of care are sorely needed. Although some preliminary evidence and guidance exists for the treatment and management of NSSI in residential settings [88] and inpatient units [46,89], evidence-based strategies remain limited. Given that psychiatric discharges in the United States for adolescents increased from 683.60 to 969.03 per 100,000 adolescents between 1996 and 2007 [90], it is important to develop evidence-based therapeutic practices for these higher levels of care. Therapeutic practices, however, should not be limited to the inpatient level of care. Given the economic pressures to limit inpatient hospitalization and shorten hospital stays, it is imperative that patients be discharged to high-quality care in the community [91]. As such, developing efficacious yet cost-effective outpatient programs that provide acute care, such as partial hospitalization and intensive outpatient programs, may be especially critical for adolescents with NSSI.

## Abbreviations

NSSI, Non-suicidal self-injury; DSH, Deliberate self-harm; CBT, Cognitive behavioral therapy; MACT, Manual-assisted cognitive-behavioral therapy; DBT, Dialectical behavior therapy; RANZCP, Royal Australian & New Zealand College of Psychiatrists; NICE, National institute for clinical excellence.

## Competing interests

The authors declare that they have no competing interests.

## Authors’ contributions

JJW synthesized the literature review and wrote the first draft. DMS and SLR completed the initial literature review. MG, KRJ, AY, and DA provided additional reviews of the literature, assisted JJW with synthesizing, and completed final drafts. All authors read and approved the final manuscript.
